# Association of dietary fiber with subjective sleep quality in hemodialysis patients: a cross-sectional study in China

**DOI:** 10.1080/07853890.2023.2176541

**Published:** 2023-02-08

**Authors:** Shuang Zhang, Shu-Xin Liu, Qi-Jun Wu, Zhi-Hong Wang, Hong Liu, Cui Dong, Ting-Ting Kuai, Lian-Lian You, Jia Xiao

**Affiliations:** a Department of Nephrology, Dalian Municipal Central Hospital, Dalian, China; b Dalian Key Laboratory of Intelligent Blood Purification, Dalian Municipal Central Hospital, Dalian, China; c Department of Clinical Epidemiology, Shengjing Hospital of China Medical University, Shenyang, China

**Keywords:** Cross-sectional studies, dietary fiber, kidney failure, chronic, renal dialysis, sleep quality

## Abstract

**Background:**

Poor sleep quality is a common problem among hemodialysis (HD) patients. Dietary fiber is a key component of a healthy diet and is beneficial for a variety of health outcomes; however, evidence of an association between dietary fiber consumption and subjective sleep quality has not been established among HD patients. Therefore, we determined the association between dietary fiber consumption and the subjective sleep quality in Chinese maintenance HD patients, taking into account fiber type and source.

**Methods:**

Dietary intake was assessed with a validated food frequency questionnaire in a cross-sectional study including 741 maintenance HD patients between December 2021 and January 2022. The daily intake of dietary fiber was categorized into three groups. The lowest tertile was used as the reference category. Sleep quality of patients was accurately calculated using the Pittsburgh sleep quality index standard questionnaire. Multivariable logistic regression model and restricted cubic spline analysis were performed to assess the relationship between dietary fiber consumption and poor sleep quality.

**Results:**

Compared with the lowest tertile group of dietary fiber intake, the highest tertile group had a lower prevalence of poor sleep quality. After adjustment for potential confounders, a higher intake of total dietary fiber (OR_tertile 3 (T3) to tertile 1 (T1)_= 0.51, 95% CI: 0.31–0.85), total insoluble dietary fiber (OR_T3 to T1_ =0.54, 95% CI: 0.33–0.89), and soluble dietary fiber in vegetables (OR_T3 to T1_ =0.61, 95% CI: 0.40–0.93) were associated with a lower prevalence of poor sleep quality. Furthermore, significant linear trends were also observed (*p* < 0.05). No significant interactions were observed in subgroup analyses.

**Conclusion:**

A higher intake of dietary fiber was inversely associated with the poor sleep quality. These findings support the current recommendations that dietary fiber is essential for health and well-being.

## Key messages

1. This study was conducted because there was not prior evidence connecting sleep quality and dietary fiber consumption in hemodialysis patients.
2. In the present study a cross-sectional design was used to assess the association between dietary fiber consumption and poor sleep quality.
3. Intake of total dietary fiber, total insoluble dietary fiber, and soluble dietary fiber in vegetables were negatively associated with poor sleep quality among maintenance hemodialysis patients.

## Introduction

Hemodialysis (HD) is the main therapy for patients with end-stage renal disease (ESRD), which leads to health problems [1]. Poor sleep quality is prevalent among HD patients, affecting 41–85% [2,3]. Poor sleep quality, as an common risk factor, affects all aspects of HD patients and predicts the quality of life [3]. Furthermore, there is a positive correlation between sleep disturbances and increased morbidity and mortality related to cardiovascular diseases and infectious complications, the two major causes of death in HD patients [4]. The precise etiology of poor sleep quality is not completely understood. Recent evidence indicates that food and nutrients may also play a part in the etiology of this disorder among patients on maintenance HD [5–7].

Dietary fiber is an essential nutrient for the human body, which is not easily digested and absorbed by the intestinum tenue, but can be fully or partially fermented in the intestinum crassum [8,9]. In general, according to the molar weight and solubility, dietary fiber can be separated into the following three sub-categories: high molar weight dietary fiber, including soluble and insoluble forms; low molar weight dietary fiber; and resistant starch [10]. Because dietary fiber is abundant in whole grains, fruits, and vegetables, it is expected to have a beneficial effect on health, such as reducing postprandial blood glucose, preventing colorectal carcinoma, reducing serum total and/or low-density lipoprotein-cholesterol levels, and reducing type 2 diabetes mellitus and cardiovascular disease risk [9,11]. Consumption of fruit and vegetables is generally discouraged on the basis of the theoretical risk of exacerbating hyperkalemia in HD patients; however, Saglimbene et al. reported that a higher consumption of these foods is associated with good outcomes among HD patients [12]. Limited evidence has shown a direct relationship between dietary intake of potassium and serum level [13]. ESRD is considered an inflammatory state. Both observational and experimental studies reported an inverse relationship between total dietary fiber intake and inflammation [14]. Inflammation has a significant role in human sleep and is positively correlated with sleep disorders [15], whereas a high-fiber diet has the potential to lower inflammation by modifying the pH and permeability of the gut [16,17], both of which are considered important biological mechanisms. Several observational studies have explored the association between dietary fiber intake and sleep quality. In a randomized-crossover inpatient study consisting of 26 normal weight adults with two 5-night phases, St-Onge and colleagues [18] reported that increased fiber intake is associated with greater deep sleep; however, a cross-sectional study that included 1002 colorectal cancer survivors performed by de Winter et al. [19] in the Netherlands indicated no association between dietary fiber intake and sleep quality. Both of these studies only focused on total fiber intake rather than the type and source, which may have an impact on the results. Therefore, we hypothesized that dietary fiber is negatively correlated with the poor sleep quality in maintenance HD patients.

To date, no studies have investigated the relationship between dietary fiber and subjective sleep quality in maintenance HD patients. Therefore, to address this important issue, we carried out a cross-sectional study to ascertain the correlation, if any, between dietary fiber and the sleep quality among maintenance HD patients. The results of the current study will help provide new dietary management advice and intervention strategies for HD patients with poor sleep quality to reduce sleep-related morbidity and mortality.

## Materials and methods

### Design and population

**Figure 1.**
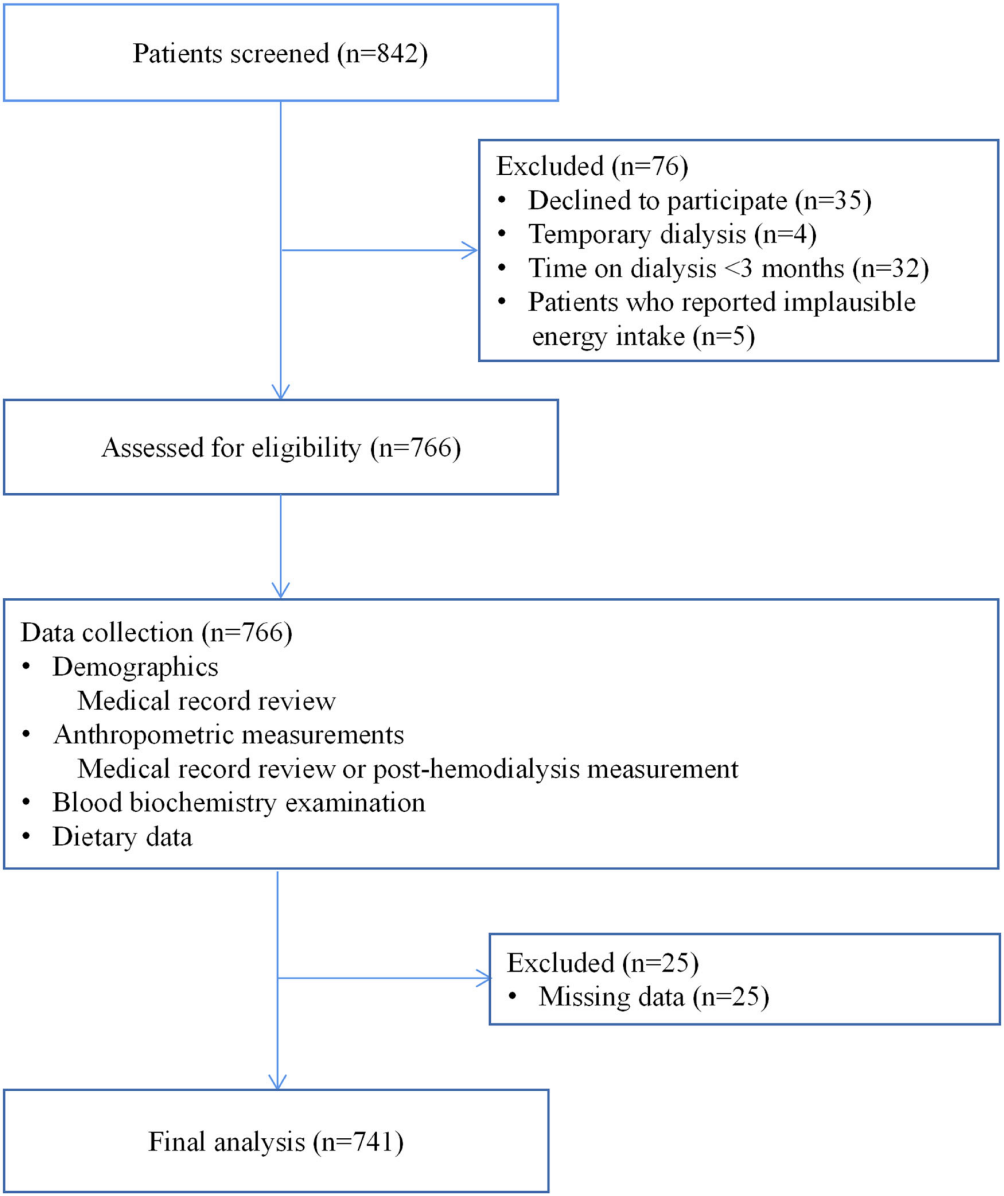
Flow chart indicates patient enrollment.

We designed a cross-sectional study that was performed from December 2021 to January 2022 and enrolled HD patients who were admitted to the Dialysis Center of Dalian Municipal Central Hospital, Dalian, China, which is the largest HD center of the three provinces in northeast China. The eligibility criteria for participants were as follows: age > 18 years; ESRD diagnosis; treatment with conventional HD for at least 3 months; ability to complete a semi-quantitative food frequency questionnaire (FFQ) and a sleep quality questionnaire; and normal intake of food and water (not receiving enteral or parenteral nutrition). We excluded HD patients who declined to participate (*n* = 35), HD patients receiving temporary dialysis (*n* = 4), time on dialysis < 3 months (*n* = 32), implausible energy intake (total energy intake not within 3 standard deviations [SDs] from the log-transformed mean [12]; *n* = 5), and those with missing variables included in the study (*n* = 25). A total of 741 maintenance HD patients were available for the final analysis (Figure 1). All study protocols conformed to the principles of the Declaration of Helsinki and were approved by the Institutional Medical Ethics Committee of Dalian Municipal Central Hospital (protocol number: YN2022–039–05). All study patients received written informed consent.

### Data collection

Baseline features were extracted from the electronic medical records of Dalian Municipal Central Hospital, including gender, age, duration of dialysis, and co-morbidities (diabetes mellitus, hypertension, and a history of cardiovascular disease [CVD]) within 1 month after enrollment. Other confounding factors were gathered by researchers according to standard operating procedures, including body mass index (BMI), education level, annual family income, physical activity, smoking status (at least 1 time per day for > 6 months), alcohol consumption (at least 1 time per day for > 6 months). Diabetes was defined as a history of diabetes mellitus or the use of anti-diabetic drugs. Hypertension was defined as the recording of hypertension in medical records or the use of antihypertensive drugs. CVD history was defined as previous angina pectoris, transient ischemic attack or cerebrovascular accident, congestive heart failure, myocardial infarction, or peripheral arterial disease. A physical examination was conducted after dialysis to measure the patient’s weight. Height was measured by trained staff following a standard protocol. The formula for calculating BMI is: BMI = weight (kg) divided by the square of height (m) (kg/m^2^). Physical activity was measured using the International Physical Activity Questionnaire-Short Form, which contains seven questions, the validity of which has been confirmed in HD patients [20].

At baseline, blood samples were taken from patients before HD treatment in the middle of the week. Biochemical indicators, including serum hemoglobin, albumin, calcium, phosphate, creatinine, C-reactive protein (CRP), ferritin, and urea nitrogen, were determined as per the standard protocol. Urea clearance was measured using standard methods and dialysis adequacy was calculated as follows: Kt/V = −ln(R－0.008 × t)+ (4－3.5 × R)×UF/W, where R is the ratio of post-dialysis to pre-dialysis serum urea nitrogen concentration, t is the duration of HD in hours, UF is the amount of ultrafiltration in liters during the HD session; and W is the post-dialysis weight in kg [21].

### Dietary Assessment

Dietary consumption was assessed with the 110-item FFQ in Chinese at the time of recruitment, which had been permitted for use and proved to have rational reliability and validity. The reproducibility coefficients of most food groups were greater than 0.5, and the Spearman correlation coefficients between FFQ and weighed diet records were between 0.3 and 0.7 [22]. The FFQ was self-reported during a routine HD treatment. According to the recommendations in the 4-day weight diet record, the standard food portion size was used to calculate the daily food intake, in grams. Assessment of food item consumption was determined by asking patients the following question: how often did you eat any food in the past year? Patients could choose among seven predefined responses (Hardly, twice or three times a month, once a week, twice or three times a week, four to six times a week, once a day, twice or more a day). The intake of each food in grams/day was calculated by multiplying consumption frequencies per day by fitted portion sizes (g/time). The dietary fiber intake was from all foods that contain fiber and individual food groups (cereals, vegetables, fruits and beans), but not supplements. The calculation of dietary fiber intake was derived using the China Food Composition tables [23].

### Sleep condition Assessment

The Pittsburgh sleep quality index (PSQI) questionnaire was used to evaluate each patient’s sleep quality, which had been permitted to use internationally and had been translated Chinese. The questionnaire was scored on a 4-point scale (0–3) and was divided into seven sleep quality components (subjective sleep quality, sleep latency, sleep duration, habitual sleep efficiency, sleep disorders, sleeping drug use, and daytime dysfunction) in the last month [24]. The PSQI questionnaire involved 19 self-assessment questions, which are composed to shape 7 ‘component’ scores. According to the guiding principles, the score of each item is between 0 (better) and 3 (worse) [24]. The seven component scores are then added to get a ‘global’ score, ranging from 0–21. The higher the score, the worse the overall sleep quality. Generally speaking, an overall PSQI score of > 5 showed poor sleep quality [24]. We have conducted special training for relatives or nursing staff of some special patients who cannot self-report sleep quality.

### Statistical analysis

The Kolmogorov-Smirnov statistic was used to test the normality of all continuous variables. The data are represented by the mean and SD of normal distribution continuous variables, the median and interquartile range (IQR) of skewed continuous variables, and the frequency and percentage of categorical variables. Variance analysis and Kruskal-Wallis test of continuous variables were used to determine differences. The Chi-square test was used for categorical variables. Just as several previous studies [25,26], the daily intake of dietary fiber was categorized based on a tertile distribution; the lowest tertile was used as the reference category. An unconditional multiple logistic regression model was used to estimate the odds ratio (OR) and corresponding 95% confidence interval (CI) of poor sleep quality. The first model adjusted for age (continuous, years), gender (male/female), and time on dialysis (continuous, months). The second model additionally adjusted for BMI (continuous, kg/m^2^), physical activity (continuous, metabolic equivalent of task [MET]/min/week), smoking status (never/ever), alcohol intake (never/ever), annual family income (continuous, RMB thousand yuan), education level (Junior high school or below, senior high school/secondary specialized school/junior college/university or above), co-morbidities (yes or no), total energy intake (continuous, kcal/day), albumin (g/L), Single-pool Kt/V_urea_ (spKt/V), CRP (continuous, mg/L), and creatinine (continuous, μmol/L) on the basis of the first model. The third model additionally adjusted for dietary protein intake (continuous, g/day) on the basis of the second model. The linear trend tests were carried out by allocating the median value of intake for each tertile of dietary fiber and taking this value as a continuous variable in a logistic regression model [27]. The interaction test was carried out by adding corresponding multiplication terms to the model at the same time. Subgroup analysis was also conducted on the basis of gender, age, diabetes, CVD, BMI, time on dialysis, dietary protein intake, and dietary energy intake. The dose-response relationship between dietary fiber intake and poor sleep quality was determined using a restricted cubic spline (RCS) regression model with five knots at the 5th, 25th, 50th, 75th and 95th percentiles, combined with logistic regression [28]. Gender, age, time on dialysis, BMI, physical activity, smoking status, alcohol consumption, household income, level of education, co-morbidities, albumin level, spKt/V, creatinine level, CRP level, and total energy and protein intake were included as covariates in RCS models to control for potential confounding effects. All analyses were performed using SAS (version 9.4; SAS Institute Inc., Cary, NC, USA). Statistical significance was set at a *p* < 0.05 and was based on a two-sided test.

## Results

As presented in the flowchart, in total of 803 maintenance HD patients engaged in the study, and 741 patients were included in the final analysis (Figure 1). The overall prevalence of poor sleep quality in our study population was 62.21%. Sociodemographic variables, lifestyle factors, and the clinical characteristics of the patients are demonstrated in Table 1. The average age of the patients was 60 years (SD = 14 years). Overall, 37.92% of the patients were male, 44.13% were former or current smokers, 35.22% were former or current alcohol drinkers, and 41.7% had diabetes. Patients had been treated with HD for a median of 49.7 months (IQR, 22.2–99.2 months). Patients with the highest tertile of dietary fiber more likely to engage in more physical activity and had a higher level of education, and had been treated with HD for a longer time compared with patients with a lower fiber intake.

**Table 1.** Baseline characteristics of study participants according to dietary fibre intake levels.

Characteristics	All patients	Tertiles of total fiber intake		
		T1 (< 11.52)	T2 (11.52–16.86)	T3 (≥ 16.86)
**Poor sleep quality/No. of patients**	461/741	164/247	148/247	149/247
**Demographics**				
Age (years)	60 ± 14	60 ± 14	60 ± 14	60 ± 13
Male (n, %)	281 (37.92)	101 (40.89)	85 (34.41)	95 (38.46)
Body mass index (kg/m^2^)	24.13 ± 4.16	24.18 ± 4.22	24.10 ± 3.95	24.10 ± 4.32
Physical activity (MET/min/week)	297 (0–1188)	297 (0–990)	231 (0–1386)	346.5 (0–933)
Educational level (n, %)				
Junior secondary or below	359 (48.45)	131 (53.04)	112 (45.34)	116 (46.96)
Senior high school/technical secondary school	199 (26.86)	60 (24.29)	70 (28.34)	69 (27.94)
Junior college/university or above	183 (24.70)	56 (22.67)	65 (26.32)	62 (25.10)
Annual family income (RMB thousand yuan), (n, %)				
<50	216 (29.15)	58 (23.48)	84 (34.01)	74 (29.96)
50 to <100	340 (45.88)	122 (49.39)	107 (43.32)	111 (44.94)
≥100	185 (24.97)	67 (27.13)	56 (22.67)	62 (25.10)
Current or former smoker, (n, %)	327 (44.13)	108 (43.72)	112 (45.34)	107 (43.32)
Current or former drinker, (n, %)	261 (35.22)	86 (34.82)	85 (34.41)	90 (36.44)
Co-morbid conditions, (n, %)				
Diabetes	309 (41.70)	94 (38.06)	118 (47.77)	97 (39.27)
Hypertension	629 (84.89)	211 (85.43)	207 (83.81)	211 (85.43)
CVD	457 (61.67)	148 (59.92)	157 (63.56)	152 (61.54)
**Laboratory parameters**				
Hemoglobin (g/L)	113 (105–121)	113 (106–120)	114 (106–123)	113 (104–123)
Albumin (g/L)	41.2 (39.2–43.3)	41.3 (38.9–43.4)	41.1 (39.1–43.3)	41.2 (39.3–43.2)
Calcium (mmol/L)	2.14 (2.03–2.25)	2.14 (2.03–2.25)	2.15 (2.03–2.25)	2.13 (2.04–2.24)
Phosphorus (mmol/L)	2.03 (1.66–2.44)	2.03 (1.63–2.41)	2.04 (1.76–2.44)	1.99 (1.59–2.46)
Creatinine (μmol/L)	891.47 ± 254.98	874.04 ± 248.29	908.94 ± 261.73	891.44 ± 254.59
C-reactive protein (mg/L)	3.3 (3.3–7.8)	3.30 (3.30–8.48)	3.30 (3.30–8.35)	3.30 (3.30–7.09)
Ferritin (mg/L)	97.97 (45.23–180.96)	89 (40.04–207.09)	98.79 (45.26–181.57)	101.75 (48.98–161.22)
Urea nitrogen (mmol/L)	28.30 (24.44–32.67)	27.99 (24.59–33.03)	29.04 (24.42–33.11)	27.91 (24.17–31.96)
Time on dialysis (months)	49.7 (22.2–99.2)	49.7 (21.8–89.3)	46.3 (24.1–96.6)	53.77 (20.3–109.7)
SpKt/V_urea_	1.33 (1.17–1.49)	1.33 (1.18–1.48)	1.33 (1.17–1.48)	1.33 (1.17–1.49)

Data presented as mean ± SD (standard deviation) or median (quartile1- quartile3) (continuous variables) or number (%) (categorical variables).

Abbreviations: MET, metabolic equivalents of task.

The dietary fiber intake characteristics of the study patients are shown in Table 2. Compared with patients with lower fiber intake, those with a higher intake tended to consume more dietary energy, protein, total soluble fiber intake, total insoluble dietary fiber, total dietary fiber in vegetables, soluble dietary fiber in vegetables, insoluble dietary fiber in vegetables, total dietary fiber in fruits, soluble dietary fiber in fruits, insoluble dietary fiber in fruits, total dietary fiber in cereal, soluble dietary fiber in cereal, total dietary fiber in beans, soluble dietary fiber in beans, and insoluble dietary fiber in beans, but less insoluble dietary fiber in cereals.

**Table 2.** Diet characteristics of study participants according to dietary fibre intake levels.

Characteristics	All patients	Tertiles of total fiber intake		
		T1 (< 11.52)	T2 (11.52–16.86)	T3 (≥ 16.86)
Poor sleep quality/No. of patients	461/741	164/247	148/247	149/247
Total energy intake (kcal/day)	1415.56 (1093.08–1784.47)	1020.74 (823.51–1277.46)	1415.20 (1195.66–1643.94)	1868.18 (1547.75–2232.51)
Protein intake (g/day)	58.51 (45.97–74.07)	43.93 (32.62–53.12)	58.20 (49.57–68.07)	77.71 (63.60–90.61)
Total soluble fiber intake (g/day)	4.63 (3.20–6.80)	2.78 (2.24–3.34)	4.75 (4.05–5.52)	7.49 (6.34–8.92)
Total insoluble dietary fiber (g/day)	9.25 (6.88–12.57)	5.85 (4.59–7.07)	9.26 (8.34–10.17)	14.12 (12.54–17.31)
Total dietary fiber in vegetables (g/day)	3.54 (2.14–5.19)	2.11 (1.48–3.25)	3.54 (2.56–4.64)	5.61 (3.94–8.03)
Soluble dietary fiber in vegetables(g/day)	1.39 (0.82–2.20)	0.85 (0.60–1.28)	1.41 (0.97–2.01)	2.24 (1.62–3.23)
Insoluble dietary fiber in vegetables(g/day)	2.04 (1.28–3.05)	1.24 (0.88–1.85)	2.11 (1.53–2.67)	3.31 (2.31–4.68)
Total dietary fiber in fruits (g/day)	2.78 (1.39–5.21)	1.40 (0.73–2.22)	2.80 (1.49–4.12)	6.14 (3.62–8.74)
Soluble dietary fiber in fruits (g/day)	0.79 (0.39–1.44)	0.41 (0.20–0.65)	0.78 (0.39–1.25)	1.63 (0.92–2.35)
Insoluble dietary fiber in fruits (g/day)	1.95 (0.96–3.81)	0.99 (0.53–1.57)	1.96 (1.01–3.13)	4.46 (2.59–6.37)
Total dietary fiber in cereal (g/day)	3.37 (2.34–4.69)	2.38 (1.85–3.26)	3.55 (2.52–4.76)	4.44 (3.34–5.79)
Soluble dietary fiber in cereal (g/day)	0.30 (0–0.70)	0.23 (0–0.46)	0.30 (0–1.00)	0.46 (0.14–1.14)
Insoluble dietary fiber in cereal (g/day)	2.81 (2.02–3.91)	2.16 (1.57–2.82)	2.85 (2.15–3.93)	0.46 (0.14–1.14)
Total dietary fiber in beans (g/day)	1.16 (0.67–1.92)	0.78 (0.31–1.15)	1.25 (0.79–1.94)	1.78 (1.04–3.13)
Soluble dietary fiber in beans (g/day)	0.77 (0.48–1.18)	0.64 (0.19–0.76)	0.86 (0.59–1.20)	1.13 (0.69–2.31)
Insoluble dietary fiber in beans (g/day)	0.36 (0.18–0.63)	0.21 (0.09–0.39)	0.38 (0.22–0.60)	0.59 (0.30–1.03)

Data presented as median (quartile1- quartile3) (continuous variables).

The associations between dietary fiber consumption and poor sleep quality among maintenance HD patients are described in Table 3. Based on the multivariate-adjusted model and compared with the reference group, a statistically significant negative association between the third tertile consumption of total dietary fiber and poor sleep quality was detected (OR = 0.51, 95% CI = 0.31–0.85), and the linear trend was also obvious (*p*_trend_ < 0.05). Similarly, negative associations were also observed between the third tertile consumption of total insoluble dietary fiber (OR = 0.54, 95% CI = 0.33–0.89), soluble dietary fiber in vegetables (OR = 0.61, 95% CI = 0.40–0.93), and poor sleep quality. Moreover, a similar linear trend was also observed (*p*_trend_ < 0.05). No significant associations were detected between other source dietary fiber intake and poor sleep quality.

**Table 3.** Adjusted hazard ratio (OR) and 95% confidence interval (CI) for the association between dietary fibre intake and the risk of poor sleep quality.

Variables	Good sleep quality	Poor sleep quality	Model 1^a^	Model 2^b^	Model 3^c^
	(*N* = 280)	(*N* = 461)			
**All sources**					
** Total dietary fiber** (g/day)					
** **T1 (<11.52)	83	164	1.00 (Ref)	1.00 (Ref)	1.00 (Ref)
** **T2 (11.52 to <16.86)	99	148	0.78 (0.54–1.13)	0.65 (0.43–0.98)	0.65 (0.43–0.98)
** **T3 (≥16.86)	98	149	0.76 (0.53–1.10)	0.52 (0.31–0.86)	0.51 (0.31–0.85)
** ***P* for trend			0.17	0.01	0.01
** Total soluble dietary fiber** (g/day)					
** **T1 (<3.64)	89	157	1.00 (Ref)	1.00 (Ref)	1.00 (Ref)
** **T2 (3.64 to <5.93)	94	153	0.94 (0.65–1.36)	0.87 (0.59–1.29)	0.86 (0.58–1.28)
** **T3 (≥5.93)	97	151	0.88 (0.61–1.27)	0.72 (0.45–1.16)	0.70 (0.43–1.14)
** ***P* for trend			0.51	0.18	0.15
** Total insoluble dietary fiber** (g/day)					
** **T1 (<7.68)	87	159	1.00 (Ref)	1.00 (Ref)	1.00 (Ref)
** **T2 (7.68 to <11.20)	91	156	0.94 (0.65–1.36)	0.79 (0.52–1.19)	0.79 (0.52–1.19)
** **T3 (≥11.20)	102	146	0.78 (0.54–1.13)	0.54 (0.33–0.89)	0.54 (0.33–0.89)
** ***P* for trend			0.18	0.01	0.01
**Vegetables sources**					
** Total dietary fiber in vegetables** (g/day)					
** **T1 (<2.57)	82	164	1.00 (Ref)	1.00 (Ref)	1.00 (Ref)
** **T2 (2.57 to <4.48)	105	142	0.68 (0.47–0.98)	0.67 (0.46–0.98)	0.65 (0.44–0.95)
** **T3 (≥4.48)	93	155	0.83 (0.57–1.21)	0.76 (0.50–1.14)	0.72 (0.47–1.09)
** ***P* for trend			0.48	0.25	0.18
** Soluble dietary fiber in vegetables**(g/day)					
** **T1 (<1.00)	79	167	1.00 (Ref)	1.00 (Ref)	1.00 (Ref)
** **T2 (1.00 to <1.87)	103	144	0.66 (0.46–0.96)	0.65 (0.44–0.95)	0.62 (0.42–0.91)
** **T3 (≥1.87)	98	150	0.73 (0.50–1.05)	0.65 (0.43–0.98)	0.61 (0.40–0.93)
** ***P* for trend			0.16	0.07	0.04
** Insoluble dietary fiber in vegetables**(g/day)					
** **T1 (<1.54)	85	161	1.00 (Ref)	1.00 (Ref)	1.00 (Ref)
** **T2 (1.54 to <2.61)	105	142	0.73 (0.50–1.05)	0.72 (0.49–1.05)	0.69 (0.47–1.02)
** **T3 (≥2.61)	90	158	0.91 (0.63–1.32)	0.83 (0.55–1.24)	0.79 (0.52–1.20)
** ***P* for trend			0.80	0.47	0.38
**Fruits sources**					
** Total dietary fiber in fruits** (g/day)					
** **T1 (<1.81)	96	150	1.00 (Ref)	1.00 (Ref)	1.00 (Ref)
** **T2 (1.81 to <4.07)	89	158	1.15 (0.80–1.66)	1.11 (0.75–1.63)	1.10 (0.75–1.62)
** **T3 (≥4.07)	95	153	1.06 (0.73–1.52)	1.01 (0.68–1.50)	1.01 (0.68–1.51)
** ***P* for trend			0.89	0.93	0.95
** Soluble dietary fiber in fruits** (g/day)					
** **T1 (<0.50)	102	144	1.00 (Ref)	1.00 (Ref)	1.00 (Ref)
** **T2 (0.50 to <1.13)	89	158	1.25 (0.87–1.81)	1.25 (0.85–1.82)	1.24 (0.84–1.81)
** **T3 (≥1.13)	89	159	1.30 (0.90–1.87)	1.30 (0.88–1.93)	1.30 (0.88–1.94)
** ***P* for trend			0.21	0.25	0.24
** Insoluble dietary fiber in fruits** (g/day)					
** **T1 (<1.26)	97	149	1.00 (Ref)	1.00 (Ref)	1.00 (Ref)
** **T2 (1.26 to <2.96)	88	159	1.17 (0.81–1.70)	1.13 (0.77–1.66)	1.13 (0.77–1.66)
** **T3 (≥2.96)	95	153	1.07 (0.74–1.54)	1.00 (0.68–1.50)	1.01 (0.68–1.50)
** ***P* for trend			0.87	0.89	0.91
**Cereal sources**					
** Total dietary fiber in cereals** (g/day)					
** **T1 (<2.59)	89	157	1.00 (Ref)	1.00 (Ref)	1.00 (Ref)
** **T2 (2.59 to <4.15)	94	153	0.91 (0.63–1.32)	0.83 (0.56–1.23)	0.83 (0.56–1.23)
** **T3 (≥4.15)	97	151	0.85 (0.59–1.23)	0.71 (0.45–1.11)	0.71 (0.45–1.11)
** ***P* for trend			0.40	0.14	0.14
** Soluble dietary fiber in cereals** (g/day)					
** **T1 (<0.08)	90	143	1.00 (Ref)	1.00 (Ref)	1.00 (Ref)
** **T2 (0.08 to <0.46)	77	141	1.23 (0.83–1.82)	1.25 (0.84–1.86)	1.25 (0.84–1.86)
** **T3 (≥0.46)	113	177	1.00 (0.70–1.43)	0.97 (0.66–1.40)	0.96 (0.66–1.40)
** ***P* for trend			0.70	0.57	0.56
** Insoluble dietary fiber in cereals** (g/day)					
** **T1 (<2.26)	86	160	1.00 (Ref)	1.00 (Ref)	1.00 (Ref)
** **T2 (2.26 to <3.50)	97	150	0.80 (0.55–1.16)	0.73 (0.49–1.08)	0.73 (0.49–1.08)
** **T3 (≥3.50)	97	151	0.82 (0.57–1.19)	0.66 (0.42–1.03)	0.66 (0.42–1.03)
** ***P* for trend			0.35	0.09	0.09
**Beans sources**					
** Total dietary fiber in beans** (g/day)					
** **T1 (<0.82)	85	160	1.00 (Ref)	1.00 (Ref)	1.00 (Ref)
** **T2 (0.82 to <1.54)	102	146	0.76 (0.52–1.10)	0.76 (0.52–1.11)	0.75 (0.51–1.10)
** **T3 (≥1.54)	93	155	0.90 (0.62–1.30)	0.84 (0.56–1.25)	0.81 (0.53–1.22)
** ***P* for trend			0.76	0.51	0.41
** Soluble dietary fiber in beans** (g/day)					
** **T1 (<0.64)	86	156	1.00 (Ref)	1.00 (Ref)	1.00 (Ref)
** **T2 (0.64 to <1.07)	96	155	0.90 (0.62–1.30)	0.90 (0.62–1.31)	0.88 (0.60–1.29)
** **T3 (≥1.07)	98	150	0.86 (0.59–1.24)	0.79 (0.53–1.17)	0.75 (0.49–1.14)
** ***P* for trend			0.42	0.24	0.17
** Insoluble dietary fiber in beans** (g/day)					
** **T1 (<0.22)	75	155	1.00 (Ref)	1.00 (Ref)	1.00 (Ref)
** **T2 (0.22 to <0.54)	109	154	0.68 (0.47–0.98)	0.68 (0.46–0.99)	0.67 (0.45–0.98)
** **T3 (≥0.54)	96	152	0.78 (0.53–1.14)	0.74 (0.49–1.10)	0.71 (0.47–1.08)
** ***P* for trend			0.35	0.24	0.18

CI: confidence interval; OR: odds ratio; T: tertiles; Ref: reference.

^a^Model 1: adjusted for gender, age and time on dialysis.

^b^Model 2: adjusted for gender, age time on dialysis, body mass index, physical activity, smoking status, drinking consumption, household income, education level, comorbidities, albumin, spKt/V, creatinine, C-reactive protein and total energy intake.

^c^Model 3: adjusted for gender, age time on dialysis, body mass index, physical activity, smoking status, drinking consumption, household income, education level, comorbidities, albumin, spKt/V, creatinine, C-reactive protein, total energy and protein intake.

Stratified analyses were carried out to evaluate the relationship between dietary fiber intake and the poor sleep quality in different subgroups. We found no variable that significantly modified the correlation between dietary fiber intake and sleep disorders for age (<60 vs. ≥60 years), gender (male vs. female), diabetes mellitus (yes vs. no), history of CVD (yes vs. no), BMI (<23 vs. ≥23 kg/m^2^), time on dialysis (<24 vs. ≥24 months), dietary fiber consumption (<1.2 vs. ≥1.2 g/kg/d), and dietary energy intake (<30 vs. ≥30 kcal/kg/d), as shown in Table 4 and Supplementary Tables 1–4 (all values of *p* for interaction > 0.05).

**Table 4.** Stratified analyses for adjusted hazard ratio (OR) and 95% confidence interval (CI) for the association between dietary fibre intake and the risk of poor sleep quality.

Characteristics	Tertiles of dietary fiber intake											
	Total dietary fiber (g/day)				Total soluble dietary fiber (g/day)				Total insoluble dietary fiber (g/day)			
	T1	T2	T3	*P_interaction_*	T1	T2	T3	*P_interaction_*	T1	T2	T3	*P_interaction_*
**Age (years)**				0.43				0.88				0.30
≤ 60	1.00 (Ref)	0.81 (0.44–1.47)	0.44 (0.20–0.94)		1.00 (Ref)	1.19 (0.67–2.10)	0.79 (0.39–1.58)		1.00 (Ref)	0.86 (0.47–1.57)	0.47 (0.22–1.00)	
> 60	1.00 (Ref)	0.59 (0.32–1.07)	0.60 (0.29–1.27)		1.00 (Ref)	0.72 (0.40–1.28)	0.60 (0.29–1.21)		1.00 (Ref)	0.82 (0.45–1.51)	0.64 (0.31–1.30)	
**Sex**				0.37				0.32				0.39
Male	1.00 (Ref)	0.80 (0.48–1.34)	0.64 (0.33–1.24)		1.00 (Ref)	1.13 (0.68–1.87)	0.97 (0.52–1.80)		1.00 (Ref)	0.96 (0.57–1.63)	0.59 (0.31–1.10)	
Female	1.00 (Ref)	0.60 (0.29–1.25)	0.41 (0.17–0.98)		1.00 (Ref)	0.68 (0.33–1.41)	0.50 (0.21–1.12)		1.00 (Ref)	0.64 (0.30–1.34)	0.48 (0.19–1.15)	
**Diabetes**				0.99				0.88				0.93
yes	1.00 (Ref)	0.94 (0.49–1.80)	0.53 (0.23–1.21)		1.00 (Ref)	1.15 (0.62–2.13)	0.88 (0.41–1.88)		1.00 (Ref)	0.87 (0.45–1.66)	0.50 (0.22–1.13)	
no	1.00 (Ref)	0.52 (0.29–0.91)	0.49 (0.24–0.96)		1.00 (Ref)	0.75 (0.43–1.31)	0.61 (0.32–1.16)		1.00 (Ref)	0.72 (0.40–1.26)	0.53 (0.27–1.03)	
**CVD**				0.31				0.28				0.29
yes	1.00 (Ref)	0.73 (0.43–1.23)	0.63 (0.33–1.21)		1.00 (Ref)	0.74 (0.44–1.22)	0.88 (0.48–1.63)		1.00 (Ref)	0.96 (0.56–1.62)	0.63 (0.33–1.20)	
no	1.00 (Ref)	0.60 (0.29–1.24)	0.30 (0.12–0.72)		1.00 (Ref)	1.38 (0.68–2.83)	0.43 (0.18–0.98)		1.00 (Ref)	0.55 (0.26–1.15)	0.32 (0.13–0.76)	
**BMI (kg/m^2^)**				0.99				0.61				0.85
< 23	1.00 (Ref)	0.49 (0.25–0.93)	0.83 (0.37–1.86)		1.00 (Ref)	0.82 (0.43–1.56)	0.89 (0.42–1.89)		1.00 (Ref)	0.65 (0.34–1.24)	0.79 (0.36–1.74)	
≥ 23	1.00 (Ref)	0.82 (0.47–1.44)	0.35 (0.17–0.70)		1.00 (Ref)	0.95 (0.55–1.61)	0.58 (0.30–1.11)		1.00 (Ref)	0.97 (0.55–1.71)	0.40 (0.20–0.78)	
**Time on dialysis (months)**				0.53				0.55				0.56
< 24	1.00 (Ref)	0.56 (0.23–1.33)	1.12 (0.38–3.30)		1.00 (Ref)	1.41 (0.61–3.25)	0.90 (0.32–2.50)		1.00 (Ref)	0.92 (0.37–2.27)	0.69 (0.24–1.96)	
≥ 24	1.00 (Ref)	0.63 (0.38–1.03)	0.37 (0.20–0.69)		1.00 (Ref)	0.78 (0.49–1.25)	0.68 (0.39–1.20)		1.00 (Ref)	0.70 (0.43–1.14)	0.44 (0.24–0.79)	
**DPI (g/kg/d)**				0.96				0.87				0.73
< 1.2	1.00 (Ref)	0.62 (0.40–0.97)	0.35 (0.19–0.63)		1.00 (Ref)	0.82 (0.54–1.26)	0.56 (0.32–0.95)		1.00 (Ref)	0.79 (0.50–1.25)	0.40 (0.23–0.70)	
≥ 1.2	1.00 (Ref)	0.54 (0.07–3.35)	0.99 (0.14–6.03)		1.00 (Ref)	1.69 (0.33–8.39)	1.53 (0.33–6.73)		1.00 (Ref)	0.67 (0.08–4.24)	1.08 (0.12–6.89)	
**DEI (kcal/kg/d)**				0.93				0.88				0.68
< 30	1.00 (Ref)	0.69 (0.44–1.06)	0.45 (0.26–0.78)		1.00 (Ref)	0.87 (0.57–1.32)	0.72 (0.42–1.21)		1.00 (Ref)	0.84 (0.54–1.30)	0.50 (0.30–0.82)	
≥ 30	1.00 (Ref)	0.02 (<0.01–0.35)	0.08 (<0.01–1.21)		1.00 (Ref)	0.32 (0.04–1.94)	0.31 (0.04–1.62)		1.00 (Ref)	0.14 (<0.05–1.50)	0.37 (0.01–3.95)	

DPI: dietary protein intake; DEI: dietary energy intake; T: tertiles; Ref: reference.

Adjusted for gender, age time on dialysis, body mass index, physical activity, smoking status, drinking consumption, household income, education level, diabetes, hypertension, cardiovascular diseases, albumin, spKt/V, creatinine, C-reactive protein, total energy and protein intake.

**Figure 2.**
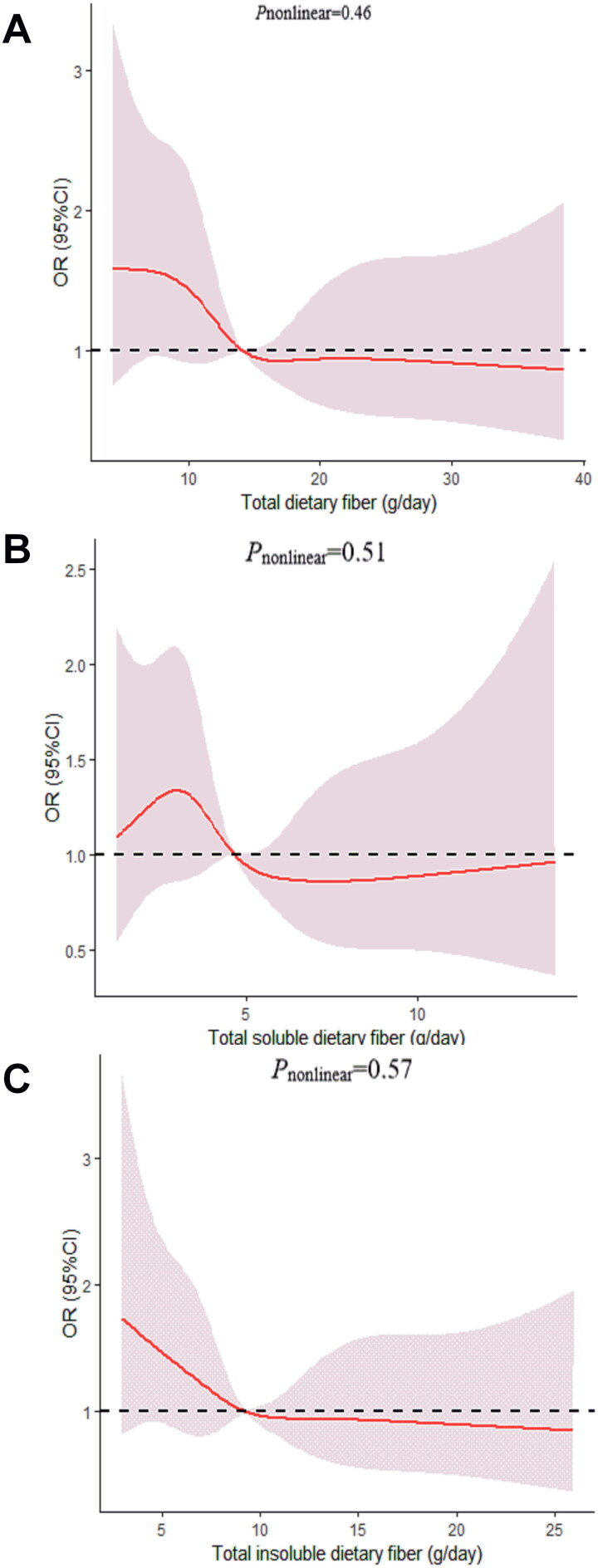
The dose-response curve of the relationship between total dietary fiber (A), total soluble dietary fiber (B), and total insoluble dietary fiber (C) consumption and poor sleep quality. The red line and shaded area represent the estimated ORs and the 95% confidence intervals. The black horizontal short, dashed line represents reference line *y* = 1.

**Figure 3.**
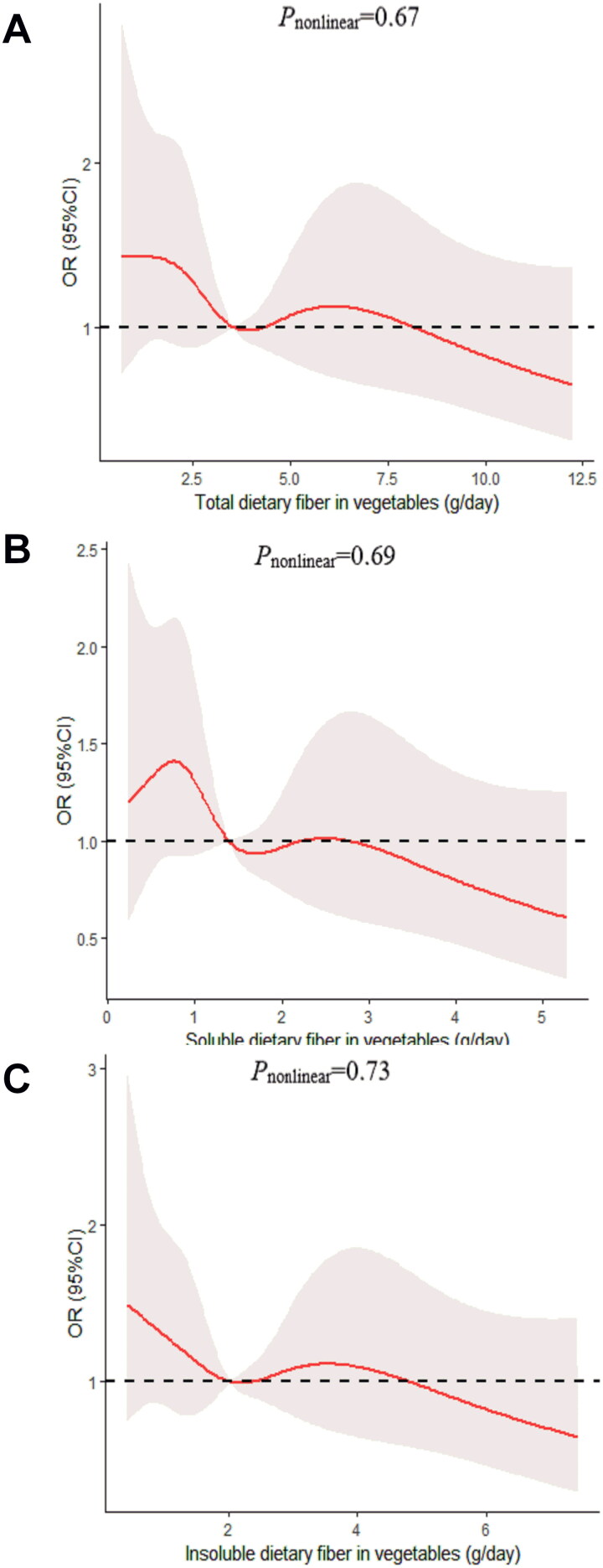
The dose-response curve of the relationship between total dietary fiber in vegetables (A), soluble dietary fiber in vegetables (B), and insoluble dietary fiber in vegetables (C) consumption and poor sleep quality. The red line and shaded area represent the estimated ORs and the 95% confidence intervals. The black horizontal short, dashed line represents reference line *y* = 1.

**Figure 4.**
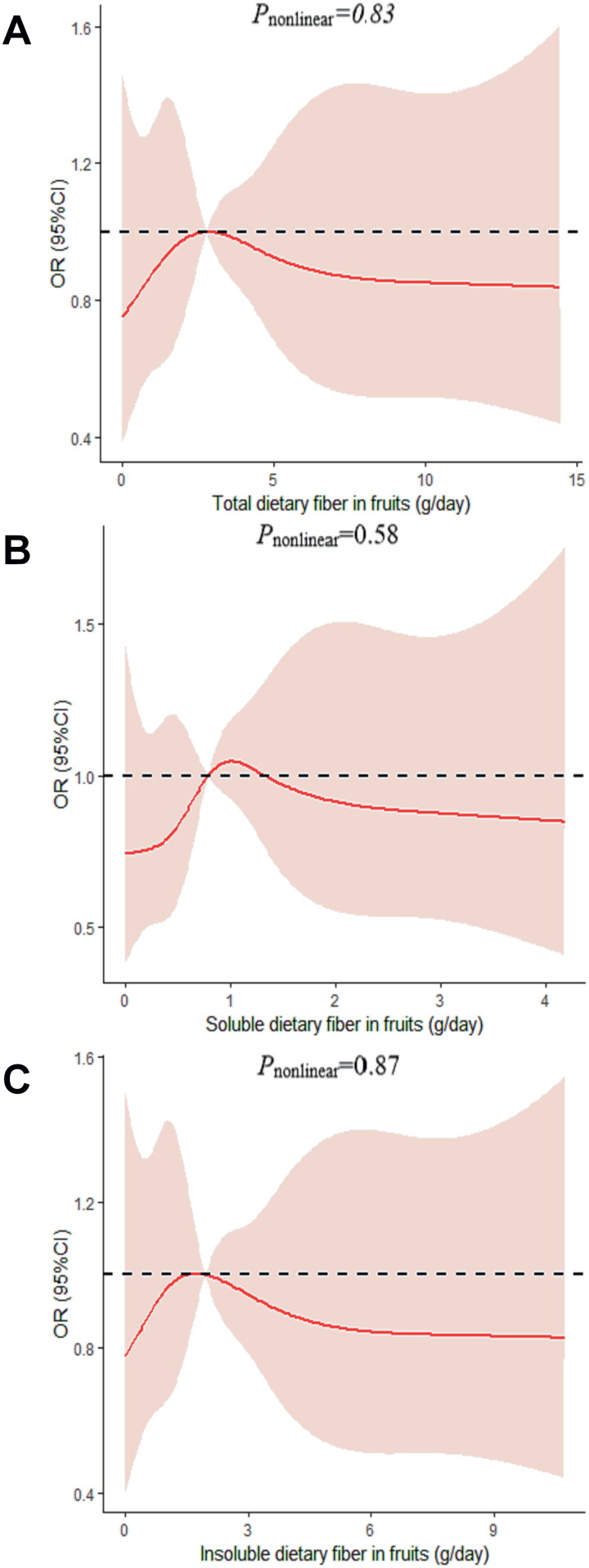
The dose-response curve of the relationship between total dietary fiber in fruits (A), soluble dietary fiber in fruits (B), and insoluble dietary fiber in fruits (C) consumption and poor sleep quality. The red line and shaded area represent the estimated ORs and the 95% confidence intervals. The black horizontal short, dashed line represents reference line *y* = 1.

**Figure 5.**
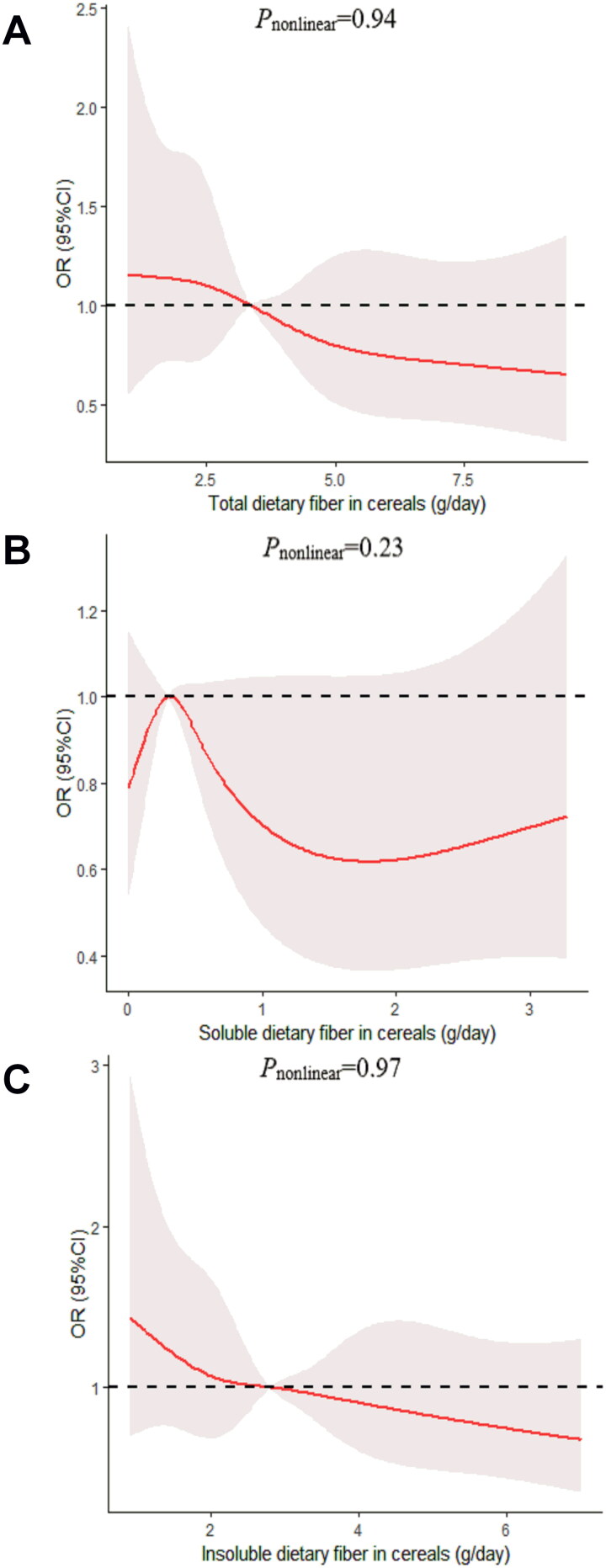
The dose-response curve of the relationship between total dietary fiber in cereals (A), soluble dietary fiber in cereals (B), and insoluble dietary fiber in cereals (C) consumption and poor sleep quality. The red line and shaded area represent the estimated ORs and the 95% confidence intervals. The black horizontal short, dashed line represents reference line *y* = 1.

**Figure 6.**
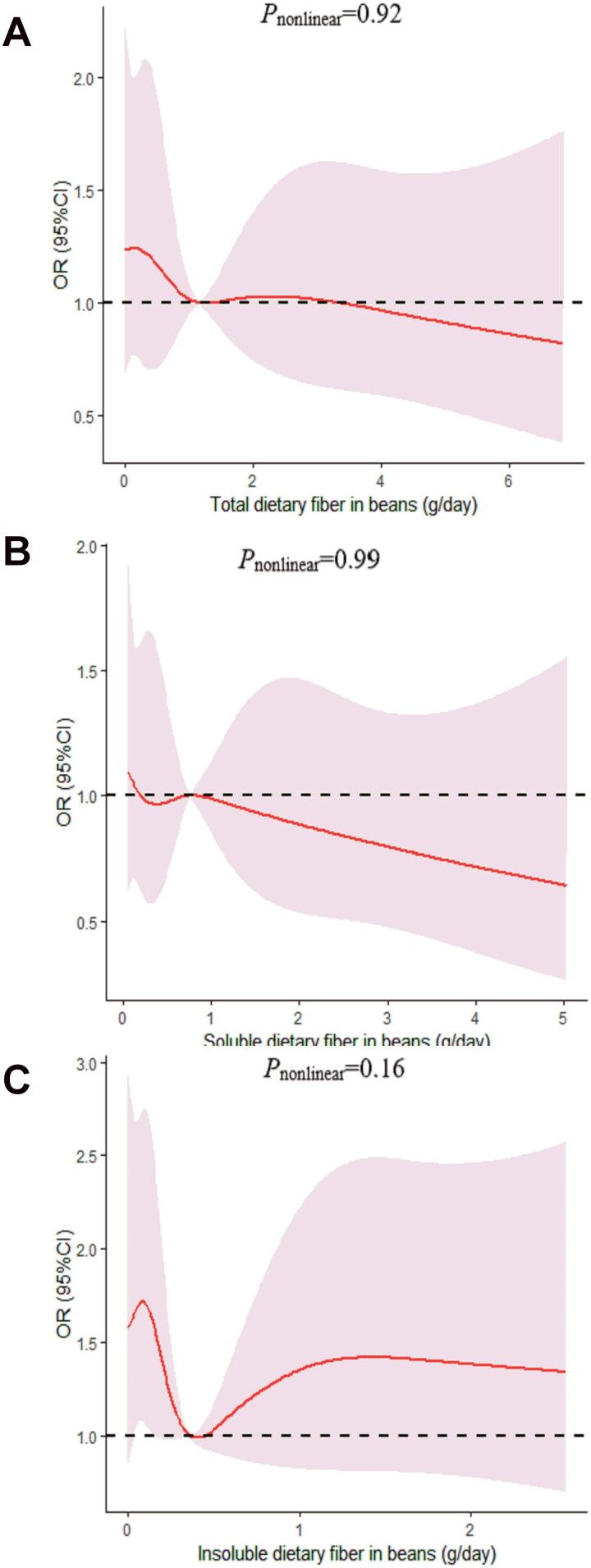
The dose-response curve of the relationship between total dietary fiber in beans (A), soluble dietary fiber in beans (B), and insoluble dietary fiber in beans (C) consumption and poor sleep quality. The red line and shaded area represent the estimated ORs and the 95% confidence intervals. The black horizontal short, dashed line represents reference line *y* = 1.

We determined the dose-response relationship between dietary fiber consumption and poor sleep quality. No significant non-linear relationship was demonstrated for dietary fiber consumption and the poor sleep quality (*p_nonlinear_* > 0.05; Figures 2–6).

## Discussion

The present findings from this cross-sectional study consisting of 741 maintenance HD patients indicated that a higher intake of total, total insoluble, and soluble dietary fiber in vegetables might improve sleep quality.

Several previous studies explored the associations between total dietary fiber intake and sleep quality in specific populations, such as healthy individuals [29], women [30], and colorectal cancer survivors [19]. However, these results have been inconsistent. In a laboratory study involving two baseline sleep nights carried out in Pennsylvania with healthy individuals 21–50 years of age, Spaeth et al. [29] reported that increased fiber consumption is associated with more slow-wave sleep when consumption was evaluated the day before and after the baseline night sleep measurement. Similarly, in a randomized-crossover inpatient study consisting of 26 normal weight adults with two 5-night phases, St-Onge et al. [18] reported that low fiber is related with lighter, less restorative sleep with more arousals. A cohort study including 4467 Mexican women conducted by Jansen et al. [30] demonstrated that the participants in the highest quartiles of the modern Mexican pattern (tortillas and soda, as well as low fiber and dairy products) were 23% more likely to have poor quality sleep than the lowest quartile. However, a cross-sectional study that included 1002 colorectal cancer survivors conducted by de Winter et al. [19] in the Netherlands showed no association between dietary fiber intake and sleep quality. As we observed, all of these studies only focused on total fiber intake rather than its type and source (e.g. soluble, insoluble, cereal, vegetable, fruit, and soy fiber), which may have different potential biological effects.

Although the biologic mechanisms explaining how dietary fiber might prevent this outcome have not been fully clarified, epidemiologic evidence indicates that the beneficial effect of high-fiber diets might be related to the effect on inflammation [31]. According to Irwin et al. [32], when sleep is disturbed, the effector system that regulates the immune system occur will change, resulting in an abnormal increase in inflammatory responses. Dietary fiber can inhibit inflammation by reducing the blood glucose load of dietary carbohydrates that can be quickly digested and absorbed [33,34]. In addition, a high-fiber diet has been related to higher plasma anti-inflammatory and adiponectin levels [35]. Furthermore, a dietary fiber-rich food might accelerate the day-night rhythm that governs temperature and increases melatonin secretion at night [18]. This finding is relevant because when melatonin levels are increased, sleep propensity and quality are highest near the falling section of the core body temperature curve [36,37]. Therefore, it is probable that fiber-rich diet might be a useful tool to improve degree of depth of sleep and structure in individuals with sleep disorders.

As our study showed, a healthier diet tends to have a lower BMI and more exercise, therefore may have less sleep apnea and better sleep quality. It has been suggested that obesity can lead to depressive mood [38], which might indirectly affect the quality of sleep. Exercise training improved global self-reported sleep quality with an effect size that was similar to that of sedative hypnotic administration in one systematic review [39]. It is believed to consist of a complex set of activities that may be physiologically and psychologically beneficial. It has been proposed that exercise training improves sleep quality through increasing energy consumption, endorphin secretion, or body temperature in a manner that facilitates sleep for recuperation of the body [40–42].

Due to the loss of renal function, hemodialysis patients are prone to hyperkalemia. Dietary recommendations for chronic kidney disease patients restrict plant-based foods, such as seeds, nuts, beans, and peas, as well as fruits and vegetables [43,44]; however, plant-based foods should not be excluded from the diet because they are also sources of vitamins, minerals, fibers, and other bioactive compounds. When prescribing a low-K^+^ diet, it is important to keep in mind that a higher intake of fibers provided by foods containing K^+^ is associated with the regulation of bowel transit, and at the very end, they may increase fecal excretion of K^+^ *via* increasing bowel motility [13,44,45].

There were several strengths in our study. First, this is the first study to examine the relationship between dietary fiber consumption and poor sleep quality among maintenance HD patients. The findings of the current study offered valuable clues for following studies on the primary prevention of sleep disorders by dietary modifications. Second, a validated FFQ was used to asses dietary information by face-to-face interviews, which provided steady estimates of dietary fiber consumption. Third, some known and suspected potential confounding factors have been adjusted for in our analyses.

Our study also had some limitations. First, this study was a cross-sectional design, so it is impossible to prove the causal relationship between dietary fiber intake and the poor sleep quality. Thus, future prospective studies require to confirm our findings. Second, dietary factors and sleep quality were assessed by self-reported methods, so recall bias is a concern. Third, we did not measure intermediate outcomes that might provide an explanatory pathway to explain the lower prevalence of poor sleep quality, nor did we measure the prevalence of hyperkalemia. Fourth, although several potential confounding variables were adjusted for in the multivariable model, we could not control the effect of unmeasured or residual confounding. Fifth, due to the limited availability of data in electronic medical record, we failed to collect information regarding antibiotic consumption. As far as we know, the dietary fiber considered as prebiotics and antibiotic intake definitely can affect the amount of probiotics as well as the effects of fiber intake that we expect. Therefore, future research should consider the impact of antibiotic use on dietary fiber intake. Finally, the patients we recruited were from one center, which made the conclusions less representative of the population of maintenance HD patients. Besides, the sample size was relatively small, which could have led to some analyses being underpowered.

In conclusion, a higher consumption of dietary fiber was shown to be have a bearing on better quality of sleep among Chinese maintenance HD patients. Though the relationships observed in this cross-sectional study need further investigation in long-term prospective studies, dietary intervention, such as increasing food diversity and an incremental increase in consumption of vegetables, might use as a possible strategy to improve sleep, especially in maintenance HD patients.

## Supplementary Material

Supplemental Material

Supplemental Material

Supplemental Material

Supplemental Material

## Data Availability

The datasets used and/or analyzed during the current study are available from the corresponding author upon reasonable request.
