# Supplementary material for: Association of dietary fiber with subjective sleep quality in hemodialysis patients: a cross-sectional study in China
Source: Ann Med. 2023 Feb 8;55(1):558–71. doi: 10.1080/07853890.2023.2176541 (PMC9930787; doi:10.1080/07853890.2023.2176541)
Supplement: Supplemental Material [file IANN_A_2176541_SM1022.doc]

**Supplementary Table 1. Stratified analyses for adjusted hazard ratio (OR) and 95% confidence interval (CI) for the association between vegetables fibre intake and the risk of poor sleep quality.**

| **Characteristics** | **Tertiles of vegetables fiber intake** | | | | | | | | | | | |
| --- | --- | --- | --- | --- | --- | --- | --- | --- | --- | --- | --- | --- |
|  | **Total dietary fiber in vegetables (g/day)** | | | | **Soluble dietary fiber in vegetables (g/day)** | | | | **Insoluble dietary fiber in vegetables (g/day)** | | | |
|  | **T_1_** | **T_2_** | **T_3_** | ***P****_interaction_* | **T_1_** | **T_2_** | **T_3_** | ***P****_interaction_* | **T_1_** | **T_2_** | **T_3_** | ***P****_interaction_* |
| **Age (years)** |  |  |  | 0.51 |  |  |  | 0.80 |  |  |  | 0.50 |
| ≤ 60 | 1.00 (Ref) | 0.80 (0.45-1.42) | 0.65 (0.34-1.20) |  | 1.00 (Ref) | 0.76 (0.42-1.36) | 0.57 (0.31-1.05) |  | 1.00 (Ref) | 0.80 (0.46-1.40) | 0.70 (0.37-1.30) |  |
| > 60 | 1.00 (Ref) | 0.52 (0.29-0.90) | 0.82 (0.45-1.51) |  | 1.00 (Ref) | 0.54 (0.31-0.95) | 0.70 (0.38-1.30) |  | 1.00 (Ref) | 0.57 (0.32-1.01) | 0.89 (0.49-1.62) |  |
| **Sex** |  |  |  | 0.49 |  |  |  | 0.30 |  |  |  | 0.64 |
| Male | 1.00 (Ref) | 1.25 (0.76-2.08) | 0.93 (0.54-1.58) |  | 1.00 (Ref) | 1.06 (0.64-1.75) | 0.76 (0.44-1.30) |  | 1.00 (Ref) | 1.20 (0.73-1.99) | 0.90 (0.53-1.55) |  |
| Female | 1.00 (Ref) | 0.22 (0.11-0.45) | 0.49 (0.22-1.05) |  | 1.00 (Ref) | 0.29 (0.14-0.57) | 0.49 (0.23-1.05) |  | 1.00 (Ref) | 0.28 (0.14-0.55) | 0.62 (0.29-1.33) |  |
| **Diabetes** |  |  |  | 0.58 |  |  |  | 0.65 |  |  |  | 0.60 |
| yes | 1.00 (Ref) | 0.70 (0.37-1.28) | 0.71 (0.36-1.36) |  | 1.00 (Ref) | 0.82 (0.43-1.56) | 0.68 (0.35-1.31) |  | 1.00 (Ref) | 0.69 (0.37-1.27) | 0.71 (0.37-1.38) |  |
| no | 1.00 (Ref) | 0.66 (0.39-1.11) | 0.79 (0.45-1.40) |  | 1.00 (Ref) | 0.59 (0.35-1.00) | 0.64 (0.36-1.13) |  | 1.00 (Ref) | 0.72 (0.42-1.20) | 0.89 (0.50-1.57) |  |
| **CVD** |  |  |  | 0.08 |  |  |  | 0.17 |  |  |  | 0.06 |
| yes | 1.00 (Ref) | 0.64 (0.39-1.06) | 0.70 (0.40-1.19) |  | 1.00 (Ref) | 0.55 (0.33-0.90) | 0.65 (0.38-1.11) |  | 1.00 (Ref) | 0.74 (0.45-1.21) | 0.73 (0.42-1.25) |  |
| no | 1.00 (Ref) | 0.56 (0.28-1.12) | 0.62 (0.29-1.30) |  | 1.00 (Ref) | 0.59 (0.29-1.17) | 0.50 (0.24-1.05) |  | 1.00 (Ref) | 0.58 (0.29-1.14) | 0.74 (0.35-1.54) |  |
| **BMI (kg/m^2^)** |  |  |  | 0.11 |  |  |  | 0.22 |  |  |  | 0.11 |
| < 23 | 1.00 (Ref) | 0.47 (0.25-0.88) | 0.72 (0.37-1.41) |  | 1.00 (Ref) | 0.51 (0.27-0.95) | 0.54 (0.27-1.05) |  | 1.00 (Ref) | 0.43 (0.23-0.81) | 0.80 (0.41-1.56) |  |
| ≥ 23 | 1.00 (Ref) | 0.72 (0.43-1.20) | 0.73 (0.41-1.27) |  | 1.00 (Ref) | 0.61 (0.36-1.02) | 0.67 (0.38-1.16) |  | 1.00 (Ref) | 0.87 (0.53-1.44) | 0.81 (0.46-1.42) |  |
| **Time on dialysis (months)** |  |  |  | 0.65 |  |  |  | 0.60 |  |  |  | 0.83 |
| < 24 | 1.00 (Ref) | 0.58 (0.25-1.31) | 1.01 (0.41-2.47) |  | 1.00 (Ref) | 0.70 (0.29-1.65) | 0.83 (0.35-1.98) |  | 1.00 (Ref) | 0.68 (0.30-1.54) | 0.99 (0.41-2.40) |  |
| ≥ 24 | 1.00 (Ref) | 0.62 (0.39-0.98) | 0.63 (0.38-1.04) |  | 1.00 (Ref) | 0.53 (0.33-0.84) | 0.53 (0.32-0.87) |  | 1.00 (Ref) | 0.64 (0.40-1.02) | 0.70 (0.42-1.15) |  |
| **DPI(g/kg/d)** |  |  |  | 0.40 |  |  |  | 0.46 |  |  |  | 0.48 |
| < 1.2 | 1.00 (Ref) | 0.67 (0.44-1.00) | 0.62 (0.39-0.99) |  | 1.00 (Ref) | 0.62 (0.41-0.95) | 0.58 (0.37-0.92) |  | 1.00 (Ref) | 0.69 (0.46-1.05) | 0.66 (0.41-1.05) |  |
| ≥ 1.2 | 1.00 (Ref) | 0.32 (0.08-1.19) | 0.63 (0.18-1.96) |  | 1.00 (Ref) | 0.34 (0.08-1.27) | 0.38 (0.10-1.23) |  | 1.00 (Ref) | 0.41 (0.10-1.53) | 0.77 (0.23-2.37) |  |
| **DEI(kcal/kg/d)** |  |  |  | 0.35 |  |  |  | 0.40 |  |  |  | 0.43 |
| < 30 | 1.00 (Ref) | 0.69 (0.45-1.04) | 0.66 (0.41-1.06) |  | 1.00 (Ref) | 0.60 (0.39-0.91) | 0.60 (0.38-0.96) |  | 1.00 (Ref) | 0.71 (0.47-1.08) | 0.72 (0.44-1.15) |  |
| ≥ 30 | 1.00 (Ref) | 0.20 (0.05-0.70) | 0.58 (0.18-1.72) |  | 1.00 (Ref) | 0.44 (0.12-1.48) | 0.43 (0.13-1.30) |  | 1.00 (Ref) | 0.23 (0.06-0.84) | 0.67 (0.21-1.98) |  |

Abbreviation: DPI: dietary protein intake; DEI: dietary energy intake; T, tertiles; Ref, reference.

Adjusted for gender, age time on dialysis, body mass index, physical activity, smoking status, drinking consumption, household income, education level, diabetes, hypertension, cardiovascular diseases, albumin, spkt/v, creatinine, C-reactive protein, total energy and protein intake.
