# Supplementary material for: Association of dietary fiber with subjective sleep quality in hemodialysis patients: a cross-sectional study in China
Source: Ann Med. 2023 Feb 8;55(1):558–71. doi: 10.1080/07853890.2023.2176541 (PMC9930787; doi:10.1080/07853890.2023.2176541)
Supplement: Supplemental Material [file IANN_A_2176541_SM1019.doc]

**Supplementary Table 2. Stratified analyses for adjusted hazard ratio (OR) and 95% confidence interval (CI) for the association between fruits fibre intake and the risk of poor sleep quality.**

| **Characteristics** | **Tertiles of fruits fiber intake** | | | | | | | | | | | |
| --- | --- | --- | --- | --- | --- | --- | --- | --- | --- | --- | --- | --- |
|  | **Total dietary fiber in fruits (g/day)** | | | | **Soluble dietary fiber in fruits (g/day)** | | | | **Insoluble dietary fiber in fruits (g/day)** | | | |
|  | **T1** | **T2** | **T3** | ***P****_interaction_* | **T_1_** | **T_2_** | **T_3_** | ***P****_interaction_* | **T_1_** | **T_2_** | **T_3_** | ***P****_interaction_* |
| **Age (years)** |  |  |  | 0.98 |  |  |  | 0.56 |  |  |  | 0.77 |
| ≤ 60 | 1.00 (Ref) | 1.16 (0.66-2.06) | 1.11 (0.62-2.00) |  | 1.00 (Ref) | 1.25 (0.71-2.20) | 1.34 (0.75-2.41) |  | 1.00 (Ref) | 1.19 (0.68-2.09) | 1.12 (0.62-2.01) |  |
| > 60 | 1.00 (Ref) | 1.07 (0.61-1.88) | 0.96 (0.54-1.69) |  | 1.00 (Ref) | 1.38 (0.79-2.40) | 1.19 (0.67-2.11) |  | 1.00 (Ref) | 1.03 (0.59-1.80) | 0.96 (0.55-1.70) |  |
| **Sex** |  |  |  | 0.25 |  |  |  | 0.30 |  |  |  | 0.29 |
| Male | 1.00 (Ref) | 1.00 (0.61-1.66) | 1.13 (0.67-1.88) |  | 1.00 (Ref) | 1.08 (0.66-1.76) | 1.57 (0.94-2.62) |  | 1.00 (Ref) | 1.06 (0.64-1.76) | 1.07 (0.65-1.77) |  |
| Female | 1.00 (Ref) | 1.27 (0.66-2.47) | 0.76 (0.38-1.50) |  | 1.00 (Ref) | 1.59 (0.82-3.14) | 0.81 (0.41-1.59) |  | 1.00 (Ref) | 1.16 (0.61-2.22) | 0.86 (0.43-1.74) |  |
| **Diabetes** |  |  |  | 0.96 |  |  |  | 0.83 |  |  |  | 0.87 |
| yes | 1.00 (Ref) | 1.43 (0.78-2.61) | 1.52 (0.80-2.90) |  | 1.00 (Ref) | 1.41 (0.77-2.60) | 1.92 (1.03-3.61) |  | 1.00 (Ref) | 1.31 (0.72-2.37) | 1.45 (0.77-2.76) |  |
| no | 1.00 (Ref) | 0.97 (0.57-1.64) | 0.78 (0.45-1.33) |  | 1.00 (Ref) | 1.21 (0.72-2.05) | 0.97 (0.57-1.67) |  | 1.00 (Ref) | 1.08 (0.64-1.84) | 0.80 (0.47-1.37) |  |
| **CVD** |  |  |  | 0.31 |  |  |  | 0.09 |  |  |  | 0.33 |
| yes | 1.00 (Ref) | 1.13 (0.69-1.86) | 1.14 (0.68-1.92) |  | 1.00 (Ref) | 1.42 (0.87-2.30) | 1.51 (0.90-2.54) |  | 1.00 (Ref) | 1.12 (0.68-1.85) | 1.11 (0.66-1.85) |  |
| no | 1.00 (Ref) | 1.14 (0.59-2.23) | 0.82 (0.42-1.60) |  | 1.00 (Ref) | 1.04 (0.53-2.06) | 1.01 (0.52-1.96) |  | 1.00 (Ref) | 1.24 (0.65-2.42) | 0.87 (0.44-1.70) |  |
| **BMI (kg/m^2^)** |  |  |  | 0.69 |  |  |  | 0.32 |  |  |  | 0.56 |
| < 23 | 1.00 (Ref) | 1.31 (0.72-2.42) | 1.38 (0.73-2.63) |  | 1.00 (Ref) | 1.60 (0.87-2.96) | 1.50 (0.80-2.84) |  | 1.00 (Ref) | 1.26 (0.69-2.32) | 1.34 (0.72-2.52) |  |
| ≥ 23 | 1.00 (Ref) | 0.93 (0.55-1.57) | 0.83 (0.48-1.42) |  | 1.00 (Ref) | 0.95 (0.56-1.59) | 1.13 (0.66-1.94) |  | 1.00 (Ref) | 0.97 (0.57-1.64) | 0.85 (0.49-1.45) |  |
| **Time on dialysis (months)** |  |  |  | 0.48 |  |  |  | 0.19 |  |  |  | 0.48 |
| < 24 | 1.00 (Ref) | 1.43 (0.61-3.39) | 1.42 (0.60-3.41) |  | 1.00 (Ref) | 2.17 (0.90-5.30) | 2.63 (1.12-6.40) |  | 1.00 (Ref) | 1.42 (0.62-3.31) | 1.32 (0.56-3.11) |  |
| ≥ 24 | 1.00 (Ref) | 1.05 (0.67-1.64) | 0.93 (0.58-1.50) |  | 1.00 (Ref) | 1.09 (0.70-1.70) | 1.07 (0.67-1.72) |  | 1.00 (Ref) | 1.12 (0.71-1.76) | 0.95 (0.59-1.53) |  |
| **DPI(g/kg/d)** |  |  |  | 0.89 |  |  |  | 0.92 |  |  |  | 0.96 |
| < 1.2 | 1.00 (Ref) | 1.00 (0.66-1.54) | 0.91 (0.58-1.42) |  | 1.00 (Ref) | 1.21 (0.80-1.84) | 1.14 (0.73-1.78) |  | 1.00 (Ref) | 1.13 (0.74-1.73) | 0.93 (0.60-1.45) |  |
| ≥ 1.2 | 1.00 (Ref) | 2.40 (0.80-7.39) | 2.11 (0.72-6.25) |  | 1.00 (Ref) | 1.93 (0.63-5.97) | 2.55 (0.90-7.45) |  | 1.00 (Ref) | 1.32 (0.45-3.86) | 1.50 (0.52-4.36) |  |
| **DEI(kcal/kg/d)** |  |  |  | 0.79 |  |  |  | 0.99 |  |  |  | 0.93 |
| < 30 | 1.00 (Ref) | 0.99 (0.65-1.51) | 0.89 (0.58-1.38) |  | 1.00 (Ref) | 1.08 (0.71-1.63) | 1.13 (0.73-1.74) |  | 1.00 (Ref) | 1.08 (0.71-1.65) | 0.92 (0.60-1.42) |  |
| ≥ 30 | 1.00 (Ref) | 1.86 (0.57-6.17) | 1.91 (0.63-5.87) |  | 1.00 (Ref) | 2.58 (0.77-9.06) | 2.60 (0.85-8.31) |  | 1.00 (Ref) | 1.26 (0.38-4.12) | 1.43 (0.47-4.36) |  |

Abbreviation: DPI: dietary protein intake; DEI: dietary energy intake; T, tertiles; Ref, reference.

Adjusted for gender, age time on dialysis, body mass index, physical activity, smoking status, drinking consumption, household income, education level, diabetes, hypertension, cardiovascular diseases, albumin, spkt/v, creatinine, C-reactive protein, total energy and protein intake.
