# Supplementary material for: Association of dietary fiber with subjective sleep quality in hemodialysis patients: a cross-sectional study in China
Source: Ann Med. 2023 Feb 8;55(1):558–71. doi: 10.1080/07853890.2023.2176541 (PMC9930787; doi:10.1080/07853890.2023.2176541)
Supplement: Supplemental Material [file IANN_A_2176541_SM1012.doc]

**Supplementary Table 3. Stratified analyses for adjusted hazard ratio (OR) and 95% confidence interval (CI) for the association between cereals fibre intake and the risk of poor sleep quality.**

| **Characteristics** | **Tertiles of cereal fiber intake** | | | | | | | | | | | |
| --- | --- | --- | --- | --- | --- | --- | --- | --- | --- | --- | --- | --- |
|  | **Total dietary fiber in cereals (g/day)** | | | | **Soluble dietary fiber in cereals (g/day)** | | | | **Insoluble dietary fiber in cereals (g/day)** | | | |
|  | **T1** | **T2** | **T3** | ***P****_interaction_* | **T_1_** | **T_2_** | **T_3_** | ***P****_interaction_* | **T_1_** | **T_2_** | **T_3_** | ***P****_interaction_* |
| **Age (years)** |  |  |  | 0.63 |  |  |  | 0.62 |  |  |  | 0.97 |
| ≤ 60 | 1.00 (Ref) | 1.01 (0.57-1.79) | 0.61 (0.32-1.16) |  | 1.00 (Ref) | 1.32 (0.74-2.34) | 1.02 (0.59-1.77) |  | 1.00 (Ref) | 0.76 (0.42-1.35) | 0.55 (0.30-1.03) |  |
| > 60 | 1.00 (Ref) | 0.71 (0.40-1.25) | 1.02 (0.52-2.04) |  | 1.00 (Ref) | 1.11 (0.62-2.00) | 0.87 (0.50-1.50) |  | 1.00 (Ref) | 0.75 (0.42-1.34) | 0.97 (0.48-1.94) |  |
| **Sex** |  |  |  | 0.73 |  |  |  | 0.89 |  |  |  | 0.73 |
| Male | 1.00 (Ref) | 0.92 (0.56-1.52) | 0.83 (0.47-1.46) |  | 1.00 (Ref) | 1.61 (0.98-2.67) | 1.17 (0.71-1.92) |  | 1.00 (Ref) | 0.74 (0.45-1.20) | 0.74 (0.42-1.30) |  |
| Female | 1.00 (Ref) | 0.73 (0.36-1.48) | 0.67 (0.29-1.50) |  | 1.00 (Ref) | 0.75 (0.37-1.54) | 0.76 (0.41-1.42) |  | 1.00 (Ref) | 0.85 (0.41-1.74) | 0.71 (0.32-1.56) |  |
| **Diabetes** |  |  |  | 0.84 |  |  |  | 0.65 |  |  |  | 0.99 |
| yes | 1.00 (Ref) | 0.76 (0.40-1.41) | 0.71 (0.34-1.47) |  | 1.00 (Ref) | 1.70 (0.91-3.21) | 0.96 (0.53-1.73) |  | 1.00 (Ref) | 0.88 (0.47-1.65) | 0.73 (0.36-1.48) |  |
| no | 1.00 (Ref) | 0.88 (0.51-1.52) | 0.75 (0.41-1.38) |  | 1.00 (Ref) | 1.01 (0.58-1.74) | 1.03 (0.61-1.72) |  | 1.00 (Ref) | 0.68 (0.40-1.16) | 0.70 (0.38-1.27) |  |
| **CVD** |  |  |  | 0.73 |  |  |  | 0.49 |  |  |  | 0.58 |
| yes | 1.00 (Ref) | 1.07 (0.64-1.79) | 1.11 (0.62-2.00) |  | 1.00 (Ref) | 1.66 (1.00-2.79) | 1.30 (0.80-2.11) |  | 1.00 (Ref) | 0.77 (0.46-1.27) | 0.90 (0.50-1.60) |  |
| no | 1.00 (Ref) | 0.58 (0.29-1.15) | 0.34 (0.16-0.74) |  | 1.00 (Ref) | 0.70 (0.34-1.41) | 0.50 (0.25-0.96) |  | 1.00 (Ref) | 0.80 (0.39-1.64) | 0.44 (0.21-0.91) |  |
| **BMI (kg/m^2^)** |  |  |  | 0.42 |  |  |  | 0.70 |  |  |  | 0.68 |
| < 23 | 1.00 (Ref) | 0.71 (0.36-1.38) | 0.45 (0.21-0.95) |  | 1.00 (Ref) | 1.45 (0.72-2.95) | 0.74 (0.41-1.33) |  | 1.00 (Ref) | 1.14 (0.58-2.26) | 0.45 (0.22-0.91) |  |
| ≥ 23 | 1.00 (Ref) | 0.91 (0.54-1.54) | 0.92 (0.51-1.67) |  | 1.00 (Ref) | 1.14 (0.69-1.88) | 1.09 (0.66-1.80) |  | 1.00 (Ref) | 0.55 (0.33-0.92) | 0.94 (0.52-1.70) |  |
| **Time on dialysis (months)** |  |  |  | 0.38 |  |  |  | 0.76 |  |  |  | 0.24 |
| < 24 | 1.00 (Ref) | 1.37 (0.59-3.20) | 1.65 (0.60-4.64) |  | 1.00 (Ref) | 1.78 (0.77-4.15) | 1.38 (0.62-3.11) |  | 1.00 (Ref) | 1.21 (0.53-2.77) | 1.59 (0.65-3.99) |  |
| ≥ 24 | 1.00 (Ref) | 0.75 (0.46-1.20) | 0.56 (0.33-0.96) |  | 1.00 (Ref) | 1.10 (0.68-1.76) | 0.89 (0.57-1.39) |  | 1.00 (Ref) | 0.67 (0.41-1.08) | 0.46 (0.27-0.79) |  |
| **DPI(g/kg/d)** |  |  |  | 0.43 |  |  |  | 0.72 |  |  |  | 0.54 |
| < 1.2 | 1.00 (Ref) | 0.85 (0.56-1.31) | 0.80 (0.48-1.32) |  | 1.00 (Ref) | 1.22 (0.80-1.87) | 1.02 (0.67-1.56) |  | 1.00 (Ref) | 0.87 (0.56-1.33) | 0.75 (0.46-1.23) |  |
| ≥ 1.2 | 1.00 (Ref) | 0.27 (0.01-1.97) | 0.20 (0.01-1.37) |  | 1.00 (Ref) | 1.61 (0.43-6.70) | 0.74 (0.28-1.87) |  | 1.00 (Ref) | <0.01 (<0.01-0.15) | <0.01 (<0.01-0.20) |  |
| **DEI(kcal/kg/d)** |  |  |  | 0.55 |  |  |  | 0.78 |  |  |  | 0.64 |
| < 30 | 1.00 (Ref) | 0.88 (0.58-1.34) | 0.83 (0.51-1.34) |  | 1.00 (Ref) | 1.30 (0.85-1.98) | 1.10 (0.72-1.67) |  | 1.00 (Ref) | 0.79 (0.52-1.20) | 0.72 (0.45-1.15) |  |
| ≥ 30 | 1.00 (Ref) | 0.17 (0.01-1.33) | 0.12 (0.01-0.89) |  | 1.00 (Ref) | 0.99 (0.26-3.93) | 0.50 (0.16-1.41) |  | 1.00 (Ref) | 0.13 (0.01-0.78) | 0.16 (0.02-0.85) |  |

Abbreviation: DPI: dietary protein intake; DEI: dietary energy intake; T, tertiles; Ref, reference.

Adjusted for gender, age time on dialysis, body mass index, physical activity, smoking status, drinking consumption, household income, education level, diabetes, hypertension, cardiovascular diseases, albumin, spkt/v, creatinine, C-reactive protein, total energy and protein intake.
