# Supplementary material for: Association of dietary fiber with subjective sleep quality in hemodialysis patients: a cross-sectional study in China
Source: Ann Med. 2023 Feb 8;55(1):558–71. doi: 10.1080/07853890.2023.2176541 (PMC9930787; doi:10.1080/07853890.2023.2176541)
Supplement: Supplemental Material [file IANN_A_2176541_SM0999.doc]

**Supplementary Table 4. Stratified analyses for adjusted hazard ratio (OR) and 95% confidence interval (CI) for the association between beans fibre intake and the risk of poor sleep quality.**

| **Characteristics** | **Tertiles of beans fiber intake** | | | | | | | | | | | |
| --- | --- | --- | --- | --- | --- | --- | --- | --- | --- | --- | --- | --- |
|  | **Total dietary fiber in beans (g/day)** | | | | **Soluble dietary fiber in beans (g/day)** | | | | **Insoluble dietary fiber in beans (g/day)** | | | |
|  | **T1** | **T2** | **T3** | ***P****_interaction_* | **T_1_** | **T_2_** | **T_3_** | ***P****_interaction_* | **T_1_** | **T_2_** | **T_3_** | ***P***_interaction_ |
| **Age (years)** |  |  |  | 0.55 |  |  |  | 0.66 |  |  |  | 0.66 |
| ≤ 60 | 1.00 (Ref) | 0.63 (0.36-1.11) | 0.66 (0.36-1.20) |  | 1.00 (Ref) | 0.72 (0.41-1.26) | 0.58 (0.31-1.08) |  | 1.00 (Ref) | 0.70 (0.39-1.24) | 0.65 (0.35-1.21) |  |
| > 60 | 1.00 (Ref) | 0.77 (0.44-1.35) | 1.03 (0.56-1.90) |  | 1.00 (Ref) | 0.96 (0.55-1.65) | 0.93 (0.51-1.70) |  | 1.00 (Ref) | 0.64 (0.37-1.10) | 0.78 (0.43-1.40) |  |
| **Sex** |  |  |  | 0.38 |  |  |  | 0.41 |  |  |  | 0.36 |
| Male | 1.00 (Ref) | 1.34 (0.81-2.21) | 1.23 (0.73-2.07) |  | 1.00 (Ref) | 1.24 (0.76-2.03) | 1.11 (0.66-1.88) |  | 1.00 (Ref) | 0.81 (0.49-1.34) | 0.87 (0.51-1.47) |  |
| Female | 1.00 (Ref) | 0.24 (0.12-0.48) | 0.39 (0.18-0.84) |  | 1.00 (Ref) | 0.41 (0.21-0.80) | 0.37 (0.17-0.77) |  | 1.00 (Ref) | 0.47 (0.24-0.91) | 0.49 (0.23-1.04) |  |
| **Diabetes** |  |  |  | 0.72 |  |  |  | 0.94 |  |  |  | 0.88 |
| yes | 1.00 (Ref) | 1.14 (0.62-2.09) | 0.79 (0.42-1.50) |  | 1.00 (Ref) | 1.21 (0.67-2.19) | 0.98 (0.52-1.85) |  | 1.00 (Ref) | 0.80 (0.44-1.46) | 0.69 (0.35-1.33) |  |
| no | 1.00 (Ref) | 0.53 (0.31-0.89) | 0.71 (0.40-1.26) |  | 1.00 (Ref) | 0.66 (0.39-1.11) | 0.58 (0.32-1.03) |  | 1.00 (Ref) | 0.57 (0.33-0.96) | 0.66 (0.38-1.14) |  |
| **CVD** |  |  |  | 0.10 |  |  |  | 0.13 |  |  |  | 0.33 |
| yes | 1.00 (Ref) | 0.60 (0.36-0.98) | 0.63 (0.36-1.08) |  | 1.00 (Ref) | 0.67 (0.40-1.09) | 0.53 (0.30-0.91) |  | 1.00 (Ref) | 0.44 (0.26-0.72) | 0.61 (0.35-1.05) |  |
| no | 1.00 (Ref) | 0.97 (0.50-1.87) | 1.08 (0.54-2.16) |  | 1.00 (Ref) | 1.21 (0.63-2.35) | 1.04 (0.51-2.09) |  | 1.00 (Ref) | 1.15 (0.59-2.26) | 0.79 (0.40-1.55) |  |
| **BMI (kg/m^2^)** |  |  |  | 0.50 |  |  |  | 0.78 |  |  |  | 0.91 |
| < 23 | 1.00 (Ref) | 0.76 (0.41-1.40) | 0.83 (0.43-1.59) |  | 1.00 (Ref) | 1.05 (0.58-1.91) | 0.84 (0.43-1.64) |  | 1.00 (Ref) | 0.92 (0.50-1.70) | 0.68 (0.34-1.31) |  |
| ≥ 23 | 1.00 (Ref) | 0.69 (0.41-1.15) | 0.78 (0.44-1.38) |  | 1.00 (Ref) | 0.73 (0.44-1.22) | 0.64 (0.37-1.11) |  | 1.00 (Ref) | 0.55 (0.33-0.92) | 0.77 (0.44-1.32) |  |
| **Time on dialysis (months)** |  |  |  | 0.85 |  |  |  | 0.73 |  |  |  | 0.41 |
| < 24 | 1.00 (Ref) | 0.64 (0.28-1.46) | 1.08 (0.47-2.48) |  | 1.00 (Ref) | 1.69 (0.76-3.82) | 1.21 (0.54-2.74) |  | 1.00 (Ref) | 0.57 (0.25-1.27) | 1.55 (0.65-3.74) |  |
| ≥ 24 | 1.00 (Ref) | 0.70 (0.44-1.10) | 0.68 (0.41-1.12) |  | 1.00 (Ref) | 0.67 (0.43-1.06) | 0.60 (0.36-1.01) |  | 1.00 (Ref) | 0.63 (0.39-0.99) | 0.51 (0.31-0.83) |  |
| **DPI(g/kg/d)** |  |  |  | 0.83 |  |  |  | 0.90 |  |  |  | 0.85 |
| < 1.2 | 1.00 (Ref) | 0.79 (0.52-1.19) | 0.75 (0.48-1.19) |  | 1.00 (Ref) | 0.84 (0.56-1.27) | 0.72 (0.46-1.14) |  | 1.00 (Ref) | 0.58 (0.38-0.88) | 0.64 (0.41-1.00) |  |
| ≥ 1.2 | 1.00 (Ref) | 0.47 (0.13-1.64) | 0.76 (0.24-2.25) |  | 1.00 (Ref) | 1.40 (0.37-5.35) | 0.89 (0.30-2.44) |  | 1.00 (Ref) | 1.88 (0.55-6.44) | 1.57 (0.51-4.70) |  |
| **DEI(kcal/kg/d)** |  |  |  | 0.75 |  |  |  | 0.99 |  |  |  | 0.89 |
| < 30 | 1.00 (Ref) | 0.75 (0.49-1.13) | 0.73 (0.45-1.16) |  | 1.00 (Ref) | 0.80 (0.53-1.21) | 0.66 (0.41-1.06) |  | 1.00 (Ref) | 0.61 (0.40-0.93) | 0.60 (0.38-0.95) |  |
| ≥ 30 | 1.00 (Ref) | 0.51 (0.15-1.64) | 0.91 (0.32-2.54) |  | 1.00 (Ref) | 1.37 (0.38-5.14) | 1.14 (0.44-2.96) |  | 1.00 (Ref) | 0.86 (0.27-2.63) | 1.31 (0.42-4.07) |  |

Abbreviation: DPI: dietary protein intake; DEI: dietary energy intake; T, tertiles; Ref, reference.

Adjusted for gender, age time on dialysis, body mass index, physical activity, smoking status, drinking consumption, household income, education level, diabetes, hypertension, cardiovascular diseases, albumin, spkt/v, creatinine, C-reactive protein, total energy and protein intake.
